# Trends in IT Innovation to Build a Next Generation Bioinformatics Solution to Manage and Analyse Biological Big Data Produced by NGS Technologies

**DOI:** 10.1155/2015/904541

**Published:** 2015-06-01

**Authors:** Alexandre G. de Brevern, Jean-Philippe Meyniel, Cécile Fairhead, Cécile Neuvéglise, Alain Malpertuy

**Affiliations:** ^1^INSERM, U 1134, DSIMB, 75739 Paris, France; ^2^University Paris Diderot, Sorbonne Paris Cité, UMR-S 1134, 75739 Paris, France; ^3^Institut National de la Transfusion Sanguine (INTS), 75739 Paris, France; ^4^Laboratoire d'Excellence GR-Ex, 75739 Paris, France; ^5^Isoft, Les Algorithmes, Bâtiment Euclide, Route de l'Orme, 91190 Saint-Aubin, France; ^6^Institut de Génétique et Microbiologie, UMR 8621 CNRS-Université Paris Sud, Bât 400, UFR des Sciences, 91405 Orsay Cedex, France; ^7^INRA, UMR 1319 Micalis, 78352 Jouy-en-Josas, France; ^8^AgroParisTech, UMR Micalis, 78352 Jouy-en-Josas, France; ^9^ATRAGENE, 33-35 rue Ledru-Rollin, 94200 Ivry-sur-Seine, France

## Abstract

Sequencing the human genome began in 1994, and 10 years of work were necessary in order to provide a nearly complete sequence. Nowadays, NGS technologies allow sequencing of a whole human genome in a few days. This deluge of data challenges scientists in many ways, as they are faced with data management issues and analysis and visualization drawbacks due to the limitations of current bioinformatics tools. In this paper, we describe how the NGS Big Data revolution changes the way of managing and analysing data. We present how biologists are confronted with abundance of methods, tools, and data formats. To overcome these problems, focus on Big Data Information Technology innovations from web and business intelligence. We underline the interest of NoSQL databases, which are much more efficient than relational databases. Since Big Data leads to the loss of interactivity with data during analysis due to high processing time, we describe solutions from the Business Intelligence that allow one to regain interactivity whatever the volume of data is. We illustrate this point with a focus on the Amadea platform. Finally, we discuss visualization challenges posed by Big Data and present the latest innovations with JavaScript graphic libraries.

## 1. Introduction

The revolution that next-generation sequencing (NGS) [1] brought about in biological sciences includes an enormous leap in the amount of data generated that needs to be stored and analysed in new ways. Storage and analysis have become critical questions. Efficient visualization methods are also needed so that the human mind can comprehend some of the rules that apply to “omics” data, be they genomics, transcriptomics, or other data from high-throughput experiments. In this paper, we aim to describe how the data have evolved, with a particular focus on DNA and RNA data and how biologists are confronted with an abundance of methods, “in-house” software, or community-developed tools, and with a variety of formats of data and databases. Many such methods imply that the biologist, to some extent, becomes proficient in some aspects of computing, and this has been a hurdle that some cannot bypass. Furthermore, some biologists, who have not been trained in information technology (IT), underestimate the expertise and time needed to set up analysis tools, to calculate results, and to imagine visualization methods that are useful to the biologist.

During the first decade of this century, an early step in the simplification of the use of bioinformatics was the development of workflow management software that allows the integration of multiple bioinformatics tools [2, 3]. Their implementation made automatization and large-scale handling of data processing possible. Nonetheless, despite their efficacy, these tools do not provide satisfying solutions for the avalanche of NGS data now available. Indeed, since these tools are merely “containers” that standardize access to bioinformatics software, they cannot surpass the current limits of these types of software, which are not yet adapted to Big Data.

There is, therefore, still a need for new, interactive tools for scientists working in large projects involving different laboratories. The future, as data will continue to grow in size and complexity, lies in online analysis and storage for collaborative work, with methods that allow high interactivity with data for analysis and visualization. In this review, we will focus on IT solutions that have emerged for management and analysis of Big Data from other fields of knowledge, such as web and Business Intelligence. These have shown their usefulness in bioinformatics, and, although we are still in the early stages, they have been used in several projects, as we will describe in this review. The main points we will develop are, first, the management of data using NoSQL databases, which are much more efficient at handling Big Data than traditional databases. They are also more flexible with regards to the integration of new data, than data models, which are the basis of relational databases. The second point we will focus on concerns the problem of loss of interactivity due to the processing time of Big Data analyses. This means that alternative hypotheses cannot be realistically tested. We will describe the emergence of answers from the Business Intelligence field that allow the processing of Big Data in real time. We will also present the case of the Amadea platform, whose interface is reminiscent of software for workflow management but is a high-performance environment for data management and analysis. Finally, we will discuss visualization tools for NGS data, and the important constraints due to the sheer size of the data. Genome Browsers are the current standard, but new tools are needed in order to be able to create in real-time graphics with web interfaces. For this, we will describe the latest innovations in terms of JavaScript graphic libraries, now largely in use for the visualization of Big Data in other fields of study. We will not discuss the, nonetheless crucial, problems of calculation nor cloud computing, as we wish to focus on new software solutions in bioinformatics. We have added a glossary for specific terms.

## 2. Next-Generation Sequencing Technologies and the Big Data Revolution

In 1994, the sequencing of the human genome began [4]. It took 10 years to complete it. A first draft of the genome was published in 2001 [5], but the final version was only published in 2004 [6]. This program cost nearly $3 billion and mobilized thousands of people [7].

In 2001, sequencing technology relied on capillaries, and reads were 500–600-base long in each of the 96 parallel reactions [8]. In the end, with this type of device, it was possible to produce 115 kilo-bases (kb) in 24 hours [1]. This capillary technology has also enabled the sequencing of other reference genomes such as the mouse, rat, chicken, dog, chimpanzee, rhesus macaque, duckbill platypus, and cow [1], opening new perspectives for comparative genomics.

**Figure 1:**
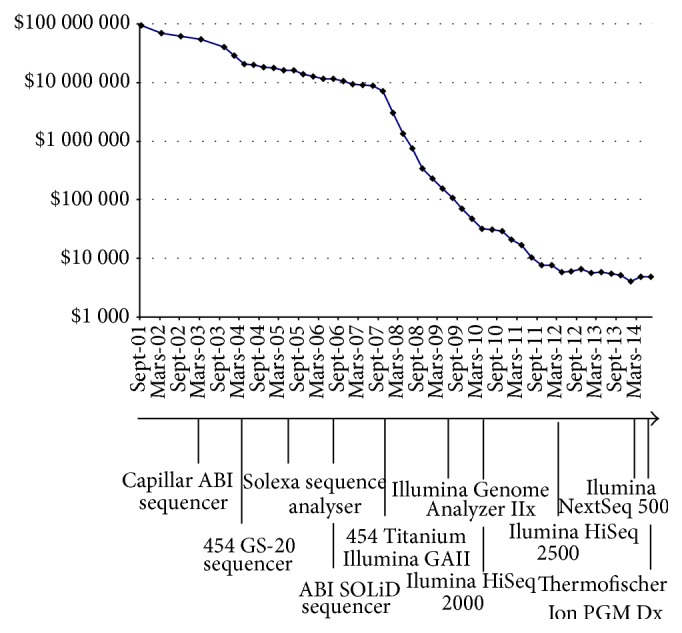
*Human genome sequencing costs*. Evolution of the costs between mid-2001 and nowadays, the different important technologies are indicated.

Around 2005, a new generation of sequencers appeared that would revolutionize the world of genomics. Sequence production capacities of these new devices allow considering, ultimately, the sequencing of entire cohorts of individuals. We had just entered the era of massive sequencing. From now on, parallel sequence runs would yield hundreds of gigabases long instead of hundred kb long. This has led to the rapid decrease of production costs, which fell much faster than expected by Moore's law (see Figure 1). Specifically, these costs were divided by a factor of 10,000 between 2004 and 2014 [9].

Today, these technologies have revolutionized NGS genomic research and are used for many applications:

- whole genome shotgun (WGS) at a population scale [10, 11]: WGS is now increasingly used in translational research such as forensic genetics [12], agrigenomics [13, 14], and clinical diagnosis. The GOLD database shows the dramatic increase in the number of genome sequences available thanks to these new technologies [15],
- rare variant discovery by whole genome resequencing or targeted sequencing,
- alternative splicing and sequence variation [16],
- ChIP-Seq [17],
- transcriptome profiling of cells, tissues or organisms,
- identification of epigenetic markers for disease diagnosis,
- many more applications (for a review, see [18]).

Such massive use of NGS technologies now generates huge amounts of data. This impressive increase of data allows new questions to be asked but also poses a number of technological and methodological challenges, as quoted many years ago in [19]. These are real bioinformatics challenges ranging from questions dealing with informatics (e.g., data storage) to experiments (e.g., quality controls), with complex algorithms, with reconstruction of genomes by mapping or assembly, and finally with genome annotation.

## 3. Bioinformatics Drawbacks

Publication in scientific journals is often subjected to the obligation for the authors to provide the produced data in a specific form for the scientific community [20]. For instance, protein structures are deposited in the Protein DataBank in standardized PDB format [21] and protein sequences in GenBank [22]. Numerous databases have been created in relation with the major ones. The fact that classical formats as pdb, fasta, or others have been created has helped tremendously the achievement of many studies.

Nonetheless, each new development has generated an impressive number of formats, without commonly accepted standards. Available tools are often not compatible and, thus, data has to undergo numerous transformations. A very representative example is the evolution of the PDB format, originally developed in the 1970s [21]; in the 1990s, crystallographers proposed an “improved” version named mmCIF [23]. The majority of users considered that it involved too many major modifications, and, as a result, it was almost never used, and new developments were done mostly with the original PDB format. Nonetheless, the original PDB format is not applicable to large complexes which are composed of many thousands of residues and base pairs, and new formats named PDBx/mmCIF will finally replace the old PDB files in 2016.

This is a typical example of how bioinformatics approaches have not evolved as fast as the technology for which they are useful. Concerning sequences, in the early 2000s, bioinformatics analyses focused on a gene or a dozen genes at most, and researchers had 227 databases [24] to obtain information about their subject of study. For these analyses, every scientist was developing small scripts and used a well-known and widespread spreadsheet software to have an overall view of their data. The scripts were executed in seconds or even minutes, and processed data volume was not constrained by the limitations of the spreadsheet software.

The problems started with microarrays. Researchers were quickly faced with a significant problem: it was no longer possible to use the spreadsheet software to visualize the data and they had to ask people with advanced computer skills to analyse the data and extract relevant information. Moreover, at that time, most machines were running with a 32-bit environment and were therefore limited to 3 billion entries in terms of RAM. With microarray data, this limit was promptly reached, for example, when a few dozen human samples were analysed. The advent of 64-bit architectures has overcome this memory problem, but other obstacles remain.

Faced with the avalanche of new data, the researchers were required to structure them in order to access and query them easily. Thus, the tool that was naturally chosen was the relational database. The major problem with this type of data structuring is that an* a priori* model of the data is required, and this, in effect, freezes them. We will see this in more detail in Section 5.1.1. In biology, concepts and technologies evolve very quickly, and new data formats appear frequently, forcing scientists to reconsider the structure of their data regularly.

Heterogeneity is also characteristic of biological data. Every institution and every machine manufacturer has developed its own data format, making unification of data even harder. This justifies even more that researchers should be knowledgeable in programming languages, in order to use existing scripts or create new ones that parse the data and extract interesting information. Indeed, many data transformation tools are available on the web. For example, there are over 200 tools for RNA-Seq ([25], including eight dedicated to differential expression analysis [26]), written in different languages and producing heterogeneous file formats, all of them covering a complete analysis cycle. The difficulties lie at the level of connecting these tools and organizing them into a routine analysis workflow and beyond, in the question of software updates and global maintenance.

Another practical example was our personal research on the imputation of the missing values in microarrays data and their influence on clustering [27]. In 2010, we tested 12 different methods implemented in 12 different ways; some being script (e.g., with R software), some being independent software, some being part of a commercial suite (e.g., MatLab), some being usable with Windows, and another only with Linux, each one with a specific (and different) format [28]. For some, we were not able to use them, even though we are proficient in bioinformatics.

In addition, the number of databases available to scientists has increased sharply from 227 databases in 2004 to 1557 in 2014 [29]. Many of these databases are novel; many have disappeared for different reasons, despite efforts by some researchers to facilitate archiving of small databases [30].

Here is the current problem which any researcher faces: how to effectively manage the heterogeneity of formats and data sources, tools, and how to extract relevant and reliable information [31].

## 4. Scientific Workflows: Advantages and Limitations

### 4.1. A Brief History of Scientific Workflows

Research has become more data-intensive and needs more computing power as large volumes of data are available from NGS technologies. This trend has led to the development of numerous tools dedicated to specific analyses and scientists usually have to combine several tools to analyse their own data. Researchers are then faced with various issues such as how to deal with access to databases, data formats, diversity of software, and scripts to use. Unfortunately, many biologists do not have sufficient relevant expertise. Hence, efforts have been made in the last two decades in order to allow scientists to take control of their data, by developing tools that facilitate access to powerful and complex computing resources.

This was initiated with the emergence of whole genome sequencing projects in the 1990s. Since the publication of the first eukaryotic chromosome, chromosome III of baker's yeast [32], and the first bacterial genome [33], there was a great need for new bioinformatics tools to access, analyse, and visualize such amounts of data. Various precursor tools of workflow management systems emerged. We can cite LASSAP [34] used in sequencing projects that was both an integrated tool for bioinformatics and a framework to integrate and combine new algorithms. This first generation of tools was primarily used by experts, since they required knowledge of the UNIX environment and command lines to perform analyses. However, with the explosion of sequence analysis needs, it became necessary to provide access to bioinformatics tools to a larger number of biologists. Many efforts were done to develop web interface to pilot bioinformatics tools and to browse databases. Then, the first tools combining web interfaces and the ability to automate treatments emerged, such as (i) The Biology Workbench [35] and (ii) PISE [36]. However, due to technological limitations, web interface interaction with the user was limited.

In parallel to the web interface development trends, standalone workflow management software was being developed to design and execute bioinformatics workflow. One of the first of these software solutions was Pipeline pilot [37] developed in 1999 by SciTegics (now part of Biovia products) for chemoinformatics and bioinformatics. Soon after, an open source project delivered the workflow management software Kepler [38]. The same year the Taverna project [39] led to the first open source platform available for bioinformaticians.

Nowadays, the typical modern workflow management software provides an IT infrastructure to easily setup, execute, and monitor a series of tasks. The aim of modern workflow management software is to facilitate integration of different software, scripts, and connection to external data sources, in order to build a data migration or analysis workflow. Modern workflow management software is usually designed to offer a rich graphical easy-to-use user interface, enabling the construction of the workflow with a “drag and drop” of the components. Such systems are based on the XML workflow language to handle complex data transformations with integration of data resources and external software.

### 4.2. Quick Overview of Some Scientific Workflow Management Software for Big Data Analysis

Several workflow management types of software are available to design scientific workflows (see Table 1). We present a summary of some that have been used to analyse NGS data.

#### 4.2.1. Galaxy

Galaxy [40–42] is an open tool dedicated to perform complex analyses in a web-oriented collaborative environment and dedicated to users with no programming skills. The Galaxy framework encapsulates high-end computational tools and provides intuitive user interface while hiding the details of computation and storage management [40]. The three objectives of Galaxy are to facilitate the accessibility and reproducibility of analysis and to assist the sharing of developed workflows [41, 42].

*Accessibility*. Galaxy users do not need to know the implementation details of the various tools. The principle is that, from a web page, users can connect to data and perform advanced genomic analysis, using tools built into the interface. To incorporate a new tool, the user has to write a configuration file that describes how to launch the tool and specifications for inputs and outputs. The approach used in Galaxy is less flexible than a programming language but it makes computation accessible for biomedical researchers averse to command lines. 

*Reproducibility*. For each analysis, a number of metadata types are generated, through which it will be possible to replicate the analysis. These include input and output datasets, tools used, and parameter values. Such types of metadata are sufficient to reproduce the analysis, but, in addition, Galaxy offers the opportunity for the user to enter annotations that should make it easier to understand the purpose of the analysis. The analysis steps are assembled in a history, in which it is possible to copy or create versions. 

*Sharing.* Galaxy has repositories where users share objects: datasets, histories, and workflows but also has Pages, elaborated web documents that enable users to communicate in detail, via texts and graphics, about their analyses.

Galaxy is implemented primarily in the Python programming language and is distributed as both a public web service and a downloadable package that can be deployed in individual laboratories.

An interesting example of Galaxy is BioBlend, a unified application programming interface (API) coded in Python language that wraps the functionality of Galaxy and CloudMan APIs [43]. BioBlend makes it possible to automate large data analysis within Galaxy using the Cloud environment of CloudMan [44].

#### 4.2.2. Kepler

We describe Kepler, despite the fact that this workflow management software has, in fact, rarely been used, up to now, in bioinformatics and NGS analysis. However, the capacity of Kepler to deal easily with distributed intensive computation infrastructures may lead to innovative development in Big Data analysis such as NGS data.

Kepler is a powerful desktop workflow management software developed primarily for ecology and environmental studies. Kepler includes a graphical user interface (GUI) for creating workflows. Kepler allows scientists to run workflows within the GUI and independently with command lines. In addition, Kepler has powerful distributed computing options to distribute workflow execution on cluster, grid, or cloud.

Recently, Kepler has been used for bioinformatics purposes. For intensive computing and NGS analysis, the bioKepler project [45] has been initiated. The bioKepler module is an extension for Kepler that integrates bioinformatics components in order to execute bioinformatics tools (e.g., BLAST [46] and HMMER [47]) and to facilitate the development of workflows for execution in distributed environments (grid platforms or cloud).

In addition, Kepler also offers the possibility to integrate the R statistics suite [48] through the RExpression component. This gives access to all R packages dedicated to NGS analysis.

#### 4.2.3. KNIME

Konstanz information miner (KNIME) is an open source workflow management software for data mining, reporting, and integration [49]. KNIME integrates various components and is used in numerous domains (e.g., Business Intelligence, customer relationship management, and marketing), including life sciences. KNIME was developed to offer a highly scalable platform. KNIME allows the easy integration of data sources (databases and files), analysis, and visualization of data. Using an intuitive graphical interface, KNIME allows users to create data analysis workflows by “drag and drop.” To enhance collaboration between research teams, KNIME allows the creation and sharing of plugins to extend its capacities. Initially used for chemoinformatics [50, 51], numerous plugins for life sciences are now available. Concerning NGS and Big Data, three extensions are proposed.

- KNIME extensions for next-generation sequencing [52] is a set of free components and workflows dedicated to NGS analysis.
- Knime4Bio [53] is a set of components for the filtering and manipulation of NGS VCF format files.
- KNIME Big Data extension [54] is dedicated to high performance access and queries on Big Data. This is a commercial extension that offers a set of components for accessing Apache Hadoop Mapreduce framework (distributed storage and distributed processing on clusters) and Apache Hive (data warehouse software for querying and managing large datasets residing in distributed storage).

In addition KNIME integrates the R statistics suite that gives access to all R packages dedicated to NGS analysis. A recent application of KNIME and R statistics was built for proteomics experiments, focusing on the screening for targets of a miRNA involved in neuroblastoma. This led to the identification of seven new gene products that were correlated with the worst clinical outcomes [55].

#### 4.2.4. Pipeline Pilot

Pipeline Pilot (Accelrys, USA) was initially developed in 1999 by SciTegics (San Diego, USA) a company that became a subsidiary of Accelrys in 2004. Professional-oriented software Pipeline Pilot is a productivity platform dealing with Big Data and one of the most commonly used workflow management types of software in pharmaceutical companies. Pipeline Pilot has visualization possibilities through the reporting component collection [56] that allow the automation of reports and the publication of web applications. In addition, Pipeline Pilot has components for integration with the visualization suite Tibco Spotfire [57], which provides powerful visualization tools and efficient interactive dashboard conception.

Pipeline Pilot has its own simple scripting language for “nonprogrammers.” This gives scientists the opportunity to build their own functional workflow prototype without needing a computer scientist at every step. Once the prototype is created, computer scientists can industrialize the workflow for routine use. This facilitates workflow development process and productivity.

Several component collections are available in many domains: biology, chemistry, material sciences, modelling and simulation, imaging, and so forth. Pipeline Pilot component collections for biology include numerous tools for sequence analysis, sequence search, or alignments such as BLAST, PSI-BLAST [58] or MegaBLAST [59], and ClustalW [60]. Specifically for NGS analysis, Accelrys proposes the next-generation sequencing collection [61] which offers powerful tools to import, analyse, and visualize NGS data. Finally, as the R statistics package is widely used by scientists, Accelrys proposes an R statistics component collection [62] to easily integrate R in the designed workflow.

#### 4.2.5. Taverna

The Taverna Workbench [39] is workflow management software created by the myGrid project. Taverna was one of the first open source workflow management types of software developed for creating scientific workflows. Taverna allows integration of SOAP and REST web services giving access to bioinformatics resources, databases, and tools [63] from numerous academic research institutes. As Taverna is widely used in academic research, a large number of components are available for scientists in various domains such as bioinformatics, chemoinformatics, medicine, astronomy, and social sciences. To enhance collaboration and workflow sharing, the myGrid consortium delivered the BioCatalogue [64], a centralized catalogue of life science web services, and myExperiment [65], a social web site for scientists, where scientists can share Taverna workflows.

One important point in Taverna is the possibility to not need to execute workflow within the Taverna Workbench. Indeed, the workflows can be run by (i) executing a command line, (ii) a remote execution server, to allow workflow running on distant machine, and (iii) through the online workflow designer and enactor OnlineHPC [38] giving access to high performance computer resources for intensive computation or Big Data analysis.

A recent example of the use of Taverna was done by the School of Computer Science at the University of Manchester. They designed and implemented a Taverna-based data refinement workflow which integrates taxonomic data retrieval, data cleaning, and data selection to define a semiautomated workflow [66].

### 4.3. Limitations

These workflow tools facilitate data analysis by noncomputer users, allowing them to control bioinformatics tools and easily analysis pipelines. They nevertheless have some limitations.

Indeed, the functional components encapsulate code written by someone who has a good knowledge of one or more programming languages (e.g., Java for KNIME, specific script for Pipeline Pilot). The different bricks are not necessarily written in the same language, and the workflow may ultimately be constrained by a brick, written in a language that is less efficient compared to other bricks. This is not a problem for* ad hoc* analyses, but as soon as one user works in a production environment, with analyses that are performed routinely, it becomes a problem.

Furthermore, if a brick is needed that does not yet exist (or a new data analysis method is needed) or if it does not match exactly the needs of the non-IT Biologist, he will not be able to intervene and he will have to seek help from developers. The power of expression of these workflow tools is more limited than that of programming languages since they do not allow immediate transcription of an idea in the environment. Researcher's exploration capacity is thus limited by the existence or not of functional bricks in the environment. The user will then be obliged to make guided data analyses and will not be free to have an entirely exploratory approach.

These environments eliminate the need to write command lines that seem obscure for noncomputer users, and they allow scientists to perform treatments, encapsulated in the different bricks, for which they will know the inputs and outputs, but ultimately users have no visibility on intermediate steps performed by the encapsulated scripts.

Furthermore, these tools do not solve one of the main issues related to NGS, namely, the volume of data that generate problems of processing. Currently, one of the preferred solutions is to move to the cloud for storage and processing of data, but this involves transferring all data to remote servers. Data transfers and admission processes in queuing can be long before the data are processed. Thus, this does not solve the problem of loss of instantaneity in obtaining results, which was observed since the advent of high dimensional data in biology.

Although these types of software simplify the use of bioinformatics tools for noncomputer scientists, they do not solve the problem of creating new intermediate components needed for a given analysis nor of rapidly exploring new hypotheses that have not previously been covered by existing encapsulated tools. Next generation bioinformatics tools have to be invented, developed, and made accessible to experts in each field of research in biology, as explained below.

## 5. Trends in IT Technologies for Big Data in Management and Analysis of NGS Data

### 5.1. Big Data and the NoSQL Revolution

#### 5.1.1. Relational Databases and Big Data

In biology, sharing data is an important issue and numerous databases are available [29]. Usually, relational database management systems are used to organize and to share biological data. Relational databases, together with web technologies, spread quickly in research institutes in the 1990s, for the diffusion of information through Internet. Relational databases meet very specific needs and are not designed to fit all scenarios. Indeed, the use of a relational database may be inappropriate under the following conditions, especially with constraints of Big Data management and sharing.

- The database cannot adapt to large traffic at an acceptable cost.
- The number of tables required to maintain the relational model rises too quickly for the corresponding amount of stored data.
- The relational model no longer meets the performance criteria because the model is no longer adapted to how the system has evolved.
- The database is subjected to a large number of temporary tables that store intermediate results.

All these limitations were noticed by major actors of the web like Amazon Inc. and Google Inc. in the 2000s. Facing the growing amount of data from the web they have undertaken to develop new classes of databases.

#### 5.1.2. NoSQL Database

The term NoSQL (“Not Only SQL”) database is a class of distributed database management systems, designed mostly for the purpose of dealing with large datasets within an acceptable timeframe for the user. This designation emerged in the second half of the 2000s with the increasing number of distributed nonrelational databases [67]. The NoSQL systems were developed in order to maintain short response time to queries, despite a very high query throughput. However, their architecture does not offer the same guarantees as relational databases (including constraints to conserve properties that guarantee that database transactions are processed reliably, namely, ACID for atomicity, consistency, isolation, and durability). In particular, the absence of a structural model for storing heterogeneous data within the same database and the necessity of rapid treatment results in the omission of some structural controls (e.g., table integrity).

Often, the transition from relational database system to a NoSQL system is motivated by several reasons such as

- a very large volume of data to store,
- frequent and massive write entries that have to be fast and reliable,
- data readings that have to be fast and consistent,
- a data model that can evolve and change on the fly (e.g., adding new concepts and data),
- ease of administration (backups and restoration),
- capability to have parallel data processing.

Existing NoSQL solutions can be grouped into four main categories.

- *Key*/*value.* This category can be viewed as a distributed hashmap. The data are simply represented by a key/value pair. The value can be a simple string, a serialized object. The interaction with the database is simple and limited to “PUT,” “GET,” and “DELETE” operations, which infers that more effort has to be put in the construction of the web application to manage complex querying. The best known solutions are Redis (Citrusbyte LLC (USA)), Riak (Basho Technologies Inc. (USA)), and Voldemort [68] created by LinkedIn.
- *Column-oriented.* This category can be viewed simply as a table in a relational database system but with a NoSQL column-oriented database, where the number of columns is dynamic. Indeed, in a relational table, the number of columns is fixed in the model and that number is the same for all records in the table. On the contrary, in a NoSQL database, the number of columns may vary from one record to another, which avoids columns with NULL values. The column-oriented NoSQL database solutions are HBase [69] an open Source implementation of the model published by Google BigTable [69] and Apache Cassandra [70], a project that respects the distributed architecture of Amazon DynamoDB (Amazon Inc. (USA)) and Google's BigTable model.
- *Oriented document.* This category is based on the key/value paradigm. The value in this case, is a JSON or XML document type. The advantage is to be able to quickly recover via a single key, a set of hierarchically structured types of information. The most popular solutions are Apache CouchDB [71], RavenDB (Hibernating Rhinos Ltd, (Israel)), and MongoDB [72].
- *Graph database.* This data representation model is based on graph theory. It is based on the notion of nodes, relationships, and properties attached to them. This model facilitates the representation of the real world, which makes it suitable for processing data in social networks. The main Graph NoSQL database solutions are InfiniteGraph (Objectivity, Inc. (USA)) and Neo4j (Neo Technology, Inc. (USA)).

Both “column-oriented” and “oriented document” categories are based on key/value systems; however the nature and structure of the value are different.

Despite their increasing deployment in several domains, the use of NoSQL databases in bioinformatics is an emerging field. NoSQL databases have caught a lot of attention in bioinformatics because of their advantages in scalability, but authors question the timeliness of their use for data organization in biology. For example, the use of graph databases was discussed by Have and Jensen [73] who concluded that “Graph queries, formulated in terms of paths, can be concise and intuitive compared to equivalent SQL queries complicated by joins.” However, the authors pointed to the limitation of actual graph query languages to enjoy the full benefits of using a graph database.

Recent use of NoSQL technologies for NGS data has been reported. Taylor [74] discussed the application of Apache Hadoop Mapreduce and the HBase NoSQL system in bioinformatics for NGS data analysis. He showed that the scalability of Big Data oriented system Hadoop and HBase is, as expected, very high. In addition, he noticed that this architecture allowed the easy integration and analysis of large and heterogeneous data sources with a small number of HBase tables. This is an important result, since, in NGS analysis, the ability to cross data with other databases is a key for discovery. Another example is the SeqWare Query engine developed by O'Connor et al. [75]. The authors use the HBase NoSQL database and deploy their application on a cloud to offer high performance for access and queries on NGS human genome data. The HBase database exhibits high performance for querying the data and, according to the authors, “enables a faster and more open exploration of results.” However, some authors tried to enhance NoSQL systems, as did Wang et al., who designed a key/value data model to support fast query over large amounts of data with HBase [76]. These authors obtained a threefold decrease of query time using NoSQL system compared to classical relational database system like Oracle.

An interesting example of a successful use of a NoSQL database is the ncRNA-DB database developed by Bonnici et al. [77]. The authors created a database to integrate ncRNA interactions data from 7 of the online data sources. The data integrated include interactions with RNA, DNA, protein, and relationship to diseases. NoSQL databases are suitable for the integration of nonstructured data and can be easily implemented for Big Data management. The authors used OrientDB, a distributed graph NoSQL database (http://www.orientechnologies.com/). Using graph NoSQL database allowed the authors to easily integrate several data sources as biological entities (genes, ncRNAs, RNAs, and diseases) and their relationships (physical interactions, functional relationships, and so on) can be modeled as a graph. The resulting graph is composed of nodes (biological entities) and edges (relations between biological entities). The ncRNA-DB offers a command line interface. The authors also developed a web interface for easily querying the database and exporting data in a text format. In addition, a Cytoscape [78] dedicated interface called ncINetView was developed to visualize the interaction graph. In this example we see that NoSQL databases offer a powerful alternative to SQL databases for Big Data integration. We further note that the representation in graph form is particularly suitable for biological data representation in a database.

Despite its scalability and performance, NoSQL is rarely used for NGS projects. The few existing examples include METEOR of INRA, used for the analysis of the impact of dietary intervention on gut microbial gene diversity [79]. However, their performance must drive scientists to pay attention to developments of NoSQL systems and to find suitable applications to overcome the limitations of relational database systems for NGS data management.

### 5.2. Innovative Big Data Analysis Technologies for NGS

#### 5.2.1. A Quick Overview of Business Intelligence Solutions

The Big Data revolution first affected major web companies (Amazon, Facebook, Google, and so forth) and large companies involved in banking and insurance, with the increasing need to extract key information from datasets in order to help decision-making processes. In IT, it is the domain of Business Intelligence that addresses the issues of decision-making. Thus, major BI solution companies offer innovative solutions to collect, to manage, and to analyse heterogeneous, large datasets. BI seeks to extend collected data and also restructure, aggregate, and reformat data in order to present them in a form that helps decision-making of the end-user. Different stakeholders are present on the market and have each developed their decision-making tools for Big Data. We can cite Cognos Business Intelligence (IBM), Reportive (CEGEDIM) [80], Oracle Business Intelligence Enterprise Edition (Oracle Corporation), and QlikView (QlikTech). All these solutions offer efficient platforms to manage and analyse Big Data. Despite their advantages, BI platforms have rarely been used in medical research and* a fortiori* on massive data sets from NGS.

However, innovative BI solutions are used by some teams involved in NGS analysis. These tools perform well and are highly beneficial to laboratories with NGS Big Data—especially those with high quality and industrialization requirements of their processes.

**Figure 2:**
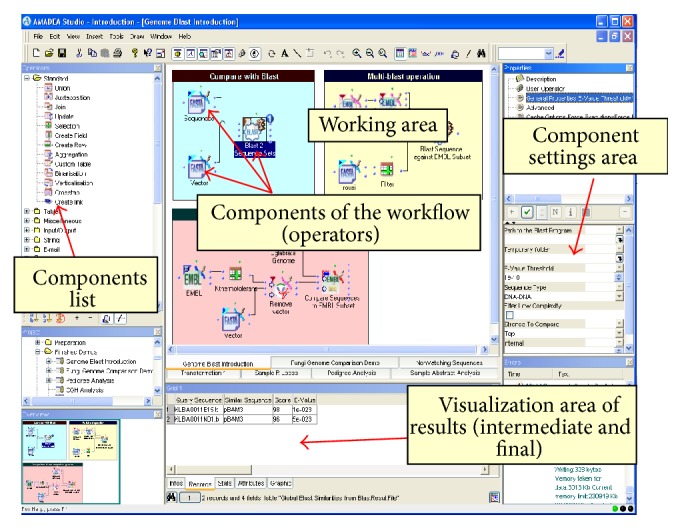
*Amadea software interface*. Users create workflow by drag and drop of components on the “working area.”

As an example of BI solution benefiting Life scientists, we focus on the successful use of the Amadea software (Isoft, France) for NGS data analysis. This is one of the first tools from Business Intelligence which has been applied in Life Science [81]. Amadea software is based on the data morphing technology, a high performance engine for data management and analysis. Data morphing is similar to image morphing that changes (or morphs) one image into another through a seamless transition. Here, data are morphed using successive processing by graphically assembling components called operators (see Figure 2). Operators are elements carrying out elementary operations (add a column to a data table, calculate the mean value, etc.) or more complex operations (call another software, compare strings, etc.). The data morphing engine allows users to access data instantaneously, whatever their size. This close real-time interaction with data offers scientist the possibility to quickly test hypotheses and find the right solution. In addition, although the Amadea software has a user interface that is similar to a workflow software, it is a true development platform, such that a scientist without special computer knowledge can develop advanced treatments and analyse Big Data sets. Thus,* in silico* work becomes highly similar to laboratory work (“wet lab”) where the results of an experiment raise new hypotheses that can be tested easily with a new analysis workflow.

The data morphing engine is developed in C++. The user interface allows the graphic elaboration of analysis workflows, by the “dragging-and-dropping” of needed components. Native components, directly linked to the engine, perform the basic operations. The combination of native components allows the creation of more sophisticated and dedicated components. The development of a new component does not require programming language knowledge. In addition, as the new component is a combination of elementary components, it takes full advantage of the data morphing engine performances. Thus, the scientist using Amadea has the same power of expression as if he was using a programming language; he has the ability to implement a missing brick in his analysis flow, without having to rely on someone else to develop it for him. This is a major difference with other existing workflow tools. This technology offers enormous flexibility for analysis. It allows one to circumvent the question of technological choices for loading and processing of the data. Indeed, in the same environment, it is possible to import, view, and compare information from relational or NoSQL database, XML, TXT, CSV, EMBL, GFF, SAM, and so forth.

**Figure 3:**
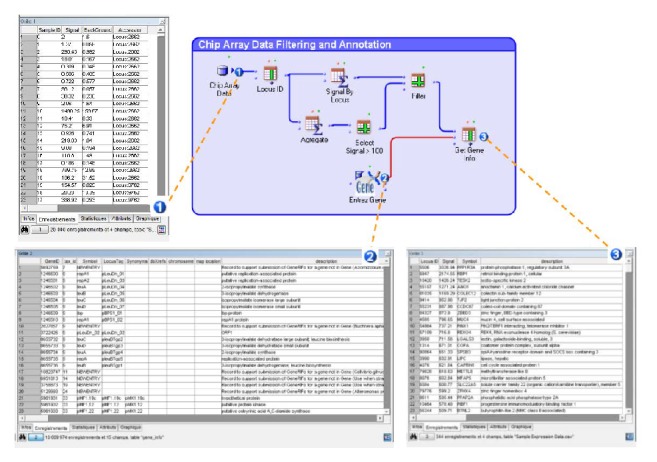
*Real-time access to all intermediate results in Amadea*. By clicking, user has instantaneous access to (1) output from the data source “chip array data,” (2) the output from “Entrez Gene” database, and (3) results from “Get Gene Info” component.

Furthermore, data are considered as “data heaps” which are displayed in tables made of rows and columns (spreadsheets). The user can use the data as he constructs his analysis workflow, without a predefined semantic. For example, in a workflow involving the Refseq databank [82], the user can select and use information about genes and, further down in the analysis process, decide to use information about proteins (see Figure 3). This flexibility is very important during exploratory phases, when it is necessary to compare data with information from various sources.

Amadea also allows the sharing, through the internet, of data as well as workflows. Online users interact with a web interface, can execute workflows, and modify parameters if needed as allowed by the programmers.

#### 5.2.2. Annotation Transfer on Yeast Genomes Using Amadea

Whereas assembly is usually included in sequencing services by sequencing companies, the structural annotation of genes, that is, the definition of gene structures including UTRs or alternatively spliced isoforms, is rarely an option and therefore remains a challenging task for many researchers. Therefore, many genomes are deposited in GenBank or EMBL/ENA without annotation or with a minimalist and often wrong annotation, which considerably limits their usefulness to researchers.

Very early in the genomics era, gene predictors were developed, providing a fast and easy means to identify genes in assembled DNA sequences from bacterial genomes, such as the GeneMark programs that have been used since 1995 [33, 71, 83]. This type of* ab initio* gene predictors uses mathematical models to identify genes. These models are specific to the sequenced genome and cannot be easily applied to other genomes and even less to eukaryotic genomes.

A next generation of genome annotation tools used EST, protein and RNA-seq aligners, and assemblers as well as combiners in addition to predictors, to improve the accuracy of their predictions (see [84], for a review on annotation tools). Genome annotation pipelines have been proposed, such as MAKER [72] or SABIA [85], as well as web prokaryote annotation servers (RAST server at the National Microbial Pathogen Data resource [86] and the MicroScope platform [87]), but this requires intensive computation resources (indeed, a full run of MAKER can take as long as a few weeks for a prokaryote genome). Computation time is an even larger problem for eukaryotic genome annotation.

Today, the typical size of a FastQ file for a yeast genome (a 10–12 Mb-long genome with a coverage of 70x) is around 2 Go. Many laboratories, such as the INRA Micalis laboratory where one of us works, are sequencing large numbers of yeast species, leading to many genomes to annotate. Time-consuming* ab initio *annotation methods are not suitable and annotation transfer has become our preferred methodology. The following existing automatic annotation transfer tools were tested and deemed inefficient: (i) GATU, dedicated to viral genomes [88], and (ii) RATT, based on synteny conservation, which only applies to closely related genomes [89]. Therefore, the INRA Micalis team chose to use the Amadea platform for developing their own automatic annotation transfer software, which had to be able to deal with significant differences between genomes. First developed for yeast genomes [90, 91], some of which are intron-rich, the Amadea workflow has been implemented to take into account the intron pattern [92] of the most closely related species used as a reference. This particularity increases the accuracy of exon-intron junction definition, in contrast to other tools, which only take into account intron-splicing motifs and overpredict the presence of spliceosomal introns. In addition, the workflow highlights ambiguous regions to simplify the final step of manual curation for high quality annotation. Finally, this annotation workflow allows data exportation in the EMBL file format, for visualization with third-party tools such as Artemis [93]. The resulting workflow leads to the annotation of a yeast genome in 5 to 7 hours compared to the use of classical annotation tools which take 70 to 80 hours to annotate such a genome. Thus, the laboratory now has at its disposal a high-performance tool for the annotation of yeast genomes, which is of high interest for annotation of genomes in the coming years, and should be useful to anyone needing a reliable, fast, and automated annotation process.

### 5.3. Big Data Visualization

#### 5.3.1. The Visualization Challenge

NGS is a powerful discovery tool for scientists seeking to glean new insights and concept from their data. However, the large amount of data leads to difficulties in their analysis and visualization. As visualization, in particular, plays a key role for discovery of new patterns and trends in large datasets, the lack of dedicated visualization tools is a major limitation to the interpretation of the data. Indeed, without Big Data-dedicated interactive visualization systems, much of the knowledge from NGS may go unrecognized.

Thus, data visualization is fundamental to NGS interpretation and several groups involved in NGS data analysis have developed specific tools for this (see Table 2). We will not describe these tools in detail, but the majority are limited to a specific type of visualization (e.g., genome assembling and browsing) and few allow easy integration of other data to enhance knowledge about specific genomic regions, for example. We can mention the efforts of ngs.plot [101] and Integrative Genome Browser [97] to integrate heterogeneous data sets such as gene annotation, clinical information, and phenotypic data to enhance discovery. As R is widely used in bioinformatics, we can also mention the Girafe package [95] for R/bioconductor, which is dedicated to NGS data analysis and offers some functions for data visualization. However, interactivity with R graphs is limited, despite some extensions that propose dynamic graphs in web pages [96, 106, 107]. Unfortunately, these extensions are not yet powerful enough to manage big data dynamic graphs and interactions through a web page. In addition, some commercial solutions are available that cover some aspects of visualization needs (e.g., Strand NGS [93], BioDT [78]). These tools and solutions are promising but still too rudimentary to be routinely used in an intensive NGS data analysis workflow. Few of them allow drawing a wide range of graphs. In addition, NGS technology and scientific analysis needs are changing very quickly. The design of dedicated data visualization interfaces is an important need for NGS analysis. Therefore, the use of turn-key solutions is not always appropriate as their capacities may not cover all the needs and their evolution may be long and costly.

The main issue of NGS is the huge amount of data leading to millions of dots to display. NGS and genomic data volumes are so big that it can be hard to imagine what these data look like. The size and complexity of so much data can be difficult to illustrate. In addition, as computer screens are limited to small resolution compared to the amount of data to display (common high resolution screens range from 1920 × 1200 to 2560 × 1440), users have to access tools to explore their data using many graphs and interact with graphs to speed up identification of key information.

Indeed, an important technical issue is to dynamically interact with graphs, for example, to change graph type, zoom-in and zoom-out, browse and change parameters on the fly, and instantaneously obtain new graphics. In addition, the revolution of genomics technologies and the large amount of available data leads to the increase of collaborative work. NGS analysis generally involves several scientists of different teams from different locations. These collaboration needs are covered by the development of web applications, which have to support increasing intensive capacities of data exploration. This induces the need to use web technologies to share data, analysis tools, and results through web applications available from the Internet, whether an intranet or an extranet. However, web applications have some technical limitations linked to current web browsers. Indeed, numerous challenges arise when creating a web application that includes interactive visualization for Big Data analysis. For example, web browsers cannot natively support huge interactive graphs and tables with thousands of pieces of data. Thus, the developers of web applications for NGS data analysis and visualization have to create applications supporting Big Data and scientists' increasing interactivity needs with their data, combined with the limitation and diversity of web browsers. With the increasing use of Big Data on the web, various technological solutions have emerged, including efficient, interactive visualization solutions. This is what we will illustrate in the next section.

#### 5.3.2. Overview of Some Visualization Solutions for NGS Big Data

As visualization helps decision-making and becomes an increasingly important component of analysis, the IT companies from the Business Intelligence (BI) domain have developed innovative solutions to deal with the Big Data visualization issue. These visualization solutions have become progressively more powerful and less expensive over the last decade. Software vendors involved in BI from other markets have solutions to visualize big data. We can cite Tibco Spotfire [68] or SAS [90], both of which are indeed used in life sciences and can significantly help scientists to visualize and explore their NGS data. The main advantage of these solutions is to offer powerful visualization with numerous types of graph for data representation and allow high interactivity with these data for changing parameters or zooming.

When developing web application with graphics, JavaScript graphic libraries are commonly used to display interactive graphics. Numerous developments have been undertaken to enhance the performance of such libraries. Using JavaScript libraries, users can easily interact with graphs by removing/adding or modifying series and points at any time. Among available JavaScript graphic libraries, some are widely used in Business Intelligence and Big Data visualization. Although recent efforts have been made to provide specific JavaScript graphic library for bioinformatics such as BioJS [108], we present in this review two relevant general JavaScript graphic libraries that can provide researchers with efficient solutions for the visualization and exploration of their NGS data.

Highcharts [92] is one of the most efficient JavaScript charting libraries that allows the inclusion of interactive charts in web interfaces. A free version is available for personal, school, and nonprofit organization use. Highcharts offers a large panel of charts such as line, area, bubble, box plot, error bars, spline, areaspline, column, bar, and pie. The graphics are rendered using SVG, VML, or the Canvas tag from HTML5 standard which is supported by modern web browsers such as Chrome 38+, Firefox 33+, or Internet Explorer 10+. Highcharts graphics are also supported by mobile devices such as iPhone/iPad or android smartphones and tablets. Highcharts is widely used in several industry domains to display interactive graphics included into web dashboards. Highcharts is also used for Big Data visualization and the authors present an example dealing with 1.7 million dots into a single figure including zooming capacities [96]. In addition, in combination with Asynchronous JavaScript and XML (AJAX) technology, the graphics can be updated in real time with data from the server or users.

A second impressive professional JavaScript graphic library is D3 Data-Driven Documents [109] also called D3.js. This graphic web library takes advantage of the capacities of modern browsers to allow powerful interactive visualization. D3.js is freely available under the terms of the BSD 3 license [75]. D3.js's growing popularity has led to numerous documented examples. To deal with large data, D3.js is usually combined with other tools such as Crossfilter [89], a JavaScript library that can explore large multivariate datasets in a web browser, or Zoomdata platform [110], a solution for fast analysis and visualization of Big Data. In addition, some BioJS components (Biojs.DNAContentViewer, Biojs.HeatmapViewer) include D3.js for advanced graphics and interactivity. Another use of D3.js is Circster [111], which adds circular genome drawing capacities to Galaxy.

These JavaScript graphics technologies allow efficient human-data interaction through a web browser. Scientists who want to explore their data and results should consider using them, since these technologies are now widely used in Big Data analysis and Business Intelligence fields and offer cost-effective solutions for interactive graphics.

## 6. Conclusion 

The development of next-generation sequencing-based studies and the abundance of available data is a true revolution for public and private laboratories. Several actors are involved in NGS data acquisitions, management, and analysis. It is crucial to access, share, and analyse these data with ease, in order to identify key information. This necessitates setting up consistent discipline-specific informatics platforms, which improve decision-making. These platforms are essential for NGS projects: they have to allow fast data access and easy workflow edition and sharing but also have to enable the integration of an increasing amount of data, through workflow editing and efficient visualization tools. They also have to facilitate collaborative work and to improve the analysis and quality of results in order to enhance discovery.

Today, numerous teams, private or academic, are working on improving NGS data analysis. Biomedical research and NGS data management analysis follow a similar evolution as other industries before. Indeed, since the end of the 1990s, the difficulty of access, manipulation, and analysis of large amount of heterogeneous data has been addressed by different IT companies from the web and BI fields, developing NoSQL databases, high performance analysis tools and Big Data visualization solutions. That is the reason why all NGS research actors have to take these IT developments into account. They have proven their efficiency and demonstrated their efficacy in solving the problems that biomedical research is facing (integration of data, sharing of data, etc.), since these problems have been already solved in other fields.

A next-generation sequencing solution to manage NGS data would have to meet the following requirements: (i) fast access to data through database systems such as NoSQL, (ii) a high performance analysis tool for instantaneous access to results, such as real-time data analysis solutions, and (iii) efficient and interactive Big Data-oriented visualization capacities through web interfaces, such as JavaScript graphic libraries recently developed for web and BI. An illustration of the interest that Life scientists should pay to solutions from major IT companies, is the recent proposal by Google to provide access to a beta version of an “API to store, process, explore, and share DNA sequence reads,” called Google Genomics [112]. It seems obvious that, in future years, this type of solutions will be a core component for bioinformatics analysis of NGS and Life Science Big Data.

## Glossary

*Data Warehouse.* A data warehouse refers to a database used to integrate data from several sources. In a company, data warehouses are usually used to create reports for decision making process.

*Datamart.* A data mart is a subset database of a data warehouse that is usually used for a specific need. 

*Hashmap.* In computing, a hash map (hash table) is a data structure used to implement an associative array, a structure that can map keys to values. 

*NGS.* Next-generation sequencing; this term has been used for the last 10 years to refer to the current methods of sequencing by synthesis: Solid, Illumina/Solexa, and Pyrosequencing. It is also used to refer to the methods in development: PacBio, Nanopore, and so forth.

*Relational Databases.* A relational database is a structured database according a relational model [113] to establish relationships between different data it contains. In a relational database, the data are organized into tables (rows and columns) with a unique key for each row. 

*Scientific Workflow.* In computing, a scientific workflow consists of an orchestrated execution of operations (data access, format conversion, and algorithm treatment) to analyse data. Scientific workflows are built by using Workflow Management software. 

*Temporary Table (in DB Querying).* In a database, a temporary table is created to temporarily store the results. Temporary tables are usually used to simplify a complex query which is decomposed into several simplified queries. 

*WGS (Whole Genome Sequencing).* Sequencing of a complete genome [114] is of particular interest when analysing variations in a population.

## Figures and Tables

**Table 1:** Commonly used scientific Workflow Management software.

Platform name	Initial creator	License	Bioinfo	Website
Galaxy	Emory University (USA) and Penn State University (USA)	Free ($)	+++	http://galaxyproject.org/

KDE	Inforsense (UK)	Commercial	++	http://www.inforsense.com/

Kepler	UC Davis, UC San Diego, and UC Santa Barbara (USA)	Free	+/−	http://kepler-project.org/

Knime	University of Konstanz (Germany)	Free and commercial	++	https://www.knime.org/

Pipeline Pilot	Accelrys (USA)	Commercial	+++	http://accelrys.com/

Taverna workbench	EBI (UK)	Free	+++	http://www.taverna.org.uk/

VIBE	Incogen (USA)	Commercial	++	http://www.incogen.com/

Columns “Bioinfo” show the level of use of these tools in bioinformatics. The “+++” indicates that the software is used by many teams, “++” indicates the software is starting to be used and “+/−” indicates that very few teams use this software in this field, and “−” the software has not been used yet in that field. The “$” sign indicates that some additional fees are required for access to third parties services such as cloud platform.

**Table 2:** List of free tools for NGS data visualization or including visualization components.

Software	Authors	Remarks	Project website
Artemis	Carver et al. [70]	Software for integrated visualization and computational analysis	http://www.sanger.ac.uk/resources/software/artemis/

CisGenome Browser	Jiang et al. [94]	Genomic data visualization	http://www-personal.umich.edu/~jianghui/browser/

Girafe	Toedling et al. [95]	Visualization of genome intervals with aligned reads. Required software: R/Bioconductor [96]	http://www.bioconductor.org/packages/release/bioc/html/girafe.html

IGV (Integrative Genomics Viewer)	Robinson et al. [97]	Genome browser and interactive exploration of large, integrated genomic datasets	http://www.broadinstitute.org/igv/

JBrowse	Westesson et al. [98]	Web-based genome browser	http://www.github.com/jbrowse/

MagicViewer	Hou et al. [99]	Assembly visualization and genetic variation annotation tool	http://bioinformatics.zj.cn/magicviewer/

NGSView	Arner et al. [100]	Sequence alignment editor	http://ngsview.sourceforge.net

ngs.plot	Shen et al. [101]	Mining and visualization of NGS data	https://code.google.com/p/ngsplot/

Savant	Fiume et al. [102]	Software for sequence annotation, visualization, and analysis	http://compbio.cs.toronto.edu/savant

seqMonk	Babraham Bioinformatics	Genome Browser	http://www.bioinformatics.babraham.ac.uk/projects/seqmonk/

Tablet	Milne et al. [103]	Graphical viewer for next-generation sequence assemblies and alignments	http://bioinf.scri.ac.uk/tablet

TGNet	Riba-grognuz et al. [104]	Method to evaluate genome scaffolding. Required software: Blat [105] and Cytoscape [78]	https://github.com/ksanao/TGNet

## References

[1] Mardis E. R. (2011). A decade's perspective on DNA sequencing technology. *Nature*.

[2] Luscombe N. M., Greenbaum D., Gerstein M. (2001). What is bioinformatics? An introduction and overview. *Yearbook of Medical Informatics*.

[3] Altman R. B., Miller K. S. (2011). 2010 translational bioinformatics year in review. *Journal of the American Medical Informatics Association*.

[4] Sinsheimer R. L. (1989). The Santa Cruz workshop—May 1985. *Genomics*.

[5] Venter J. C., Adams M. D., Myers E. W. (2001). The sequence of the human genome. *Science*.

[6] International Human Genome Sequencing Consortium (2004). Finishing the euchromatic sequence of the human genome. *Nature*.

[7] Lander E. S. (2011). Initial impact of the sequencing of the human genome. *Nature*.

[8] Dovichi N. J., Zhang J. (2000). How capillary electrophoresis sequenced the human genome this essay is based on a lecture given at the Analytica 2000 conference in Munich (Germany) on the occasion of the Heinrich-Emanuel-Merck Prize presentation. *Angewandte Chemie—International Edition*.

[9] Hayden E. C. (2014). Technology: The $1,000 genome. *Nature*.

[10] Abecasis G. R., Altshuler D., Auton A. (2010). A map of human genome variation from population-scale sequencing. *Nature*.

[11] Genome 10K Community of Scientists (2009). Genome 10K: a proposal to obtain whole-genome sequence for 10,000 vertebrate species. *Journal of Heredity*.

[12] Weber-Lehmann J., Schilling E., Gradl G., Richter D. C., Wiehler J., Rolf B. (2014). Finding the needle in the haystack: differentiating ‘identical’ twins in paternity testing and forensics by ultra-deep next generation sequencing. *Forensic Science International: Genetics*.

[13] Poland J. A., Brown P. J., Sorrells M. E., Jannink J.-L. (2012). Development of high-density genetic maps for barley and wheat using a novel two-enzyme genotyping-by-sequencing approach. *PLoS ONE*.

[14] Goddard M. E., Hayes B. J. (2009). Mapping genes for complex traits in domestic animals and their use in breeding programmes. *Nature Reviews Genetics*.

[15] Reddy T. B., Thomas A. D., Stamatis D. (2015). The Genomes OnLine Database (GOLD) v.5: a metadata management system based on a four level (meta)genome project classification. *Nucleic Acids Research*.

[16] Wang Z., Gerstein M., Snyder M. (2009). RNA-Seq: a revolutionary tool for transcriptomics. *Nature Reviews Genetics*.

[17] Park P. J. (2009). ChIP-seq: advantages and challenges of a maturing technology. *Nature Reviews Genetics*.

[18] van Dijk E. L., Auger H., Jaszczyszyn Y., Thermes C. (2014). Ten years of next-generation sequencing technology. *Trends in Genetics*.

[19] Pope C., Ziebland S., Mays N. (2000). Qualitative research in health care. Analysing qualitative data. *The British Medical Journal*.

[20] McGourty C. (1989). Databases: Johns Hopkins as international host. *Nature*.

[21] Bernstein F. C., Koetzle T. F., Williams G. J. B. (1977). The protein data bank. A computer-based archival file for macromolecular structures. *European Journal of Biochemistry*.

[22] Benson D. A., Karsch-Mizrachi I., Lipman D. J., Ostell J., Wheeler D. L. (2005). GenBank. *Nucleic Acids Research*.

[23] Bourne P. E., Berman H. M., McMahon B., Watenpaugh K. D., Westbrook J. D., Fitzgerald P. M. D. (1997). Macromolecular crystallographic information file. *Methods in Enzymology*.

[24] Baxevanis A. D. (2000). The molecular biology database collection: an online compilation of relevant database resources. *Nucleic Acids Research*.

[25] Wikipedia http://en.wikipedia.org/wiki/List_of_RNA-Seq_bioinformatics_tools.

[26] Seyednasrollah F., Laiho A., Elo L. L. (2015). Comparison of software packages for detecting differential expression in RNA-seq studies. *Briefings in Bioinformatics*.

[27] de Brevern A. G., Hazout S., Malpertuy A. (2004). Influence of microarrays experiments missing values on the stability of gene groups by hierarchical clustering. *BMC Bioinformatics*.

[28] Celton M., Malpertuy A., Lelandais G., de Brevern A. G. (2010). Comparative analysis of missing value imputation methods to improve clustering and interpretation of microarray experiments. *BMC Genomics*.

[29] Fernández-Suárez X. M., Rigden D. J., Galperin M. Y. (2014). The 2014 nucleic acids research database issue and an updated NAR online molecular biology database collection. *Nucleic Acids Research*.

[30] Tan T. W., Xie C., de Silva M. (2013). Simple re-instantiation of small databases using cloud computing. *BMC Genomics*.

[31] Merelli I., Pérez-Sánchez H., Gesing S., D’Agostino D. (2014). Managing, analysing, and integrating big data in medical bioinformatics: open problems and future perspectives. *BioMed Research International*.

[32] Oliver S. G., van der Aart Q. J. M., Agostoni-Carbone M. L. (1992). The complete DNA sequence of yeast chromosome III. *Nature*.

[33] Fleischmann R. D., White O., Kirkness E. F. (1995). Whole-genome random sequencing and assembly of *Haemophilus* influenzae Rd. *Science*.

[34] Glémet E., Codani J.-J. (1997). LASSAP, a large scale sequence comparison package. *Computer Applications in the Biosciences*.

[35] Subramaniam S. (1998). The biology workbench—a seamless database and analysis environment for the biologist. *Proteins: Structure, Function and Genetics*.

[36] Letondal C. (2001). A Web interface generator for molecular biology programs in Unix. *Bioinformatics*.

[37] Hassan M., Brown R. D., Varma-O'Brien S., Rogers D. (2006). Cheminformatics analysis and learning in a data pipelining environment. *Molecular Diversity*.

[38] Altintas I., Berkley C., Jaeger E., Jones M., Ludäscher B., Mock S. Kepler: an extensible system for design and execution of scientific workflows.

[39] Oinn T., Addis M., Ferris J. (2004). Taverna: a tool for the composition and enactment of bioinformatics workflows. *Bioinformatics*.

[40] Blankenberg D., von Kuster G., Coraor N. (2010). Galaxy: a web-based genome analysis tool for experimentalists. *Current Protocols in Molecular Biology*.

[41] Giardine B., Riemer C., Hardison R. C. (2005). Galaxy: a platform for interactive large-scale genome analysis. *Genome Research*.

[42] Goecks J., Nekrutenko A., Taylor J. (2010). Galaxy: a comprehensive approach for supporting accessible, reproducible, and transparent computational research in the life sciences. *Genome Biology*.

[43] Sloggett C., Goonasekera N., Afgan E. (2013). BioBlend: automating pipeline analyses within Galaxy and CloudMan. *Bioinformatics*.

[44] Leo S., Pireddu L., Cuccuru G. (2014). BioBlend.objects: metacomputing with Galaxy. *Bioinformatics*.

[45] Altintas I., Wang J., Crawl D., Li W. Challenges and approaches for distributed workflow-driven analysis of large-scale biological data.

[46] Altschul S. F., Gish W., Miller W., Myers E. W., Lipman D. J. (1990). Basic local alignment search tool. *Journal of Molecular Biology*.

[47] Krogh A., Brown M., Mian I. S., Sjolander K., Haussler D. (1994). Hidden Markov Models in computational biology: applications to protein modeling. *Journal of Molecular Biology*.

[48] Team R. C. (2013). *R: A Language and Environment for Statistical Computing*.

[49] Berthold M. R., Cebron N., Dill F. (2008). KNIME: the konstanz information miner. *Data Analysis, Machine Learning and Applications*.

[50] Beisken S., Meinl T., Wiswedel B., de Figueiredo L. F., Berthold M., Steinbeck C. (2013). KNIME-CDK: workflow-driven cheminformatics. *BMC Bioinformatics*.

[51] Warr W. A. (2012). Scientific workflow systems: Pipeline Pilot and KNIME. *Journal of Computer-Aided Molecular Design*.

[52] Jagla B., Wiswedel B., Coppée J.-Y. (2011). Extending KNIME for next-generation sequencing data analysis. *Bioinformatics*.

[53] Lindenbaum P., Le scouarnec S., Portero V., Redon R. (2011). Knime4Bio: a set of custom nodes for the interpretation of next-generation sequencing data with KNIME. *Bioinformatics*.

[54] Stransky N., Vallot C., Reyal F. (2006). Regional copy number-independent deregulation of transcription in cancer. *Nature Genetics*.

[55] de Antonellis P., Carotenuto M., Vandenbussche J. (2014). Early targets of miR-34a in neuroblastoma. *Molecular & Cellular Proteomics*.

[56] http://accelrys.com/products/pipeline-pilot/component-collections/reporting.html.

[57] http://spotfire.tibco.com/.

[58] Altschul S. F., Madden T. L., Schäffer A. A. (1997). Gapped BLAST and PSI-BLAST: a new generation of protein database search programs. *Nucleic Acids Research*.

[59] Morgulis A., Coulouris G., Raytselis Y., Madden T. L., Agarwala R., Schäffer A. A. (2008). Database indexing for production MegaBLAST searches. *Bioinformatics*.

[60] Larkin M. A., Blackshields G., Brown N. P. (2007). Clustal W and Clustal X version 2.0. *Bioinformatics*.

[61] http://accelrys.com/products/pipeline-pilot/component-collections/r-stats.html.

[62] http://www.sas.com/en_us/insights/big-data/data-visualization.html.

[63] Hull D., Wolstencroft K., Stevens R. (2006). Taverna: a tool for building and running workflows of services. *Nucleic Acids Research*.

[64] Bhagat J., Tanoh F., Nzuobontane E. (2010). BioCatalogue: a universal catalogue of web services for the life sciences. *Nucleic Acids Research*.

[65] Goble C. A., Bhagat J., Aleksejevs S. (2010). myExperiment: a repository and social network for the sharing of bioinformatics workflows. *Nucleic Acids Research*.

[66] Mathew C., Güntsch A., Obst M. (2014). A semi-automated workflow for biodiversity data retrieval, cleaning, and quality control. *Biodiversity Data Journal*.

[67] Lith A., Mattsson J. (2010). *Investigating Storage Solutions for Large Data*.

[68] http://www.project-voldemort.com/voldemort/.

[69] Chang F., Dean J., Ghemawat S. Bigtable: a distributed storage system for structured data.

[70] Carver T., Harris S. R., Berriman M., Parkhill J., McQuillan J. A. (2012). Artemis: an integrated platform for visualization and analysis of high-throughput sequence-based experimental data. *Bioinformatics*.

[71] Bult C. J., White O., Olsen G. J. (1996). Complete genome sequence of the Methanogenic archaeon, *Methanococcus jannaschii*. *Science*.

[72] Cantarel B. L., Korf I., Robb S. M. C. (2008). MAKER: an easy-to-use annotation pipeline designed for emerging model organism genomes. *Genome Research*.

[73] Have C. T., Jensen L. J. (2013). Are graph databases ready for bioinformatics?. *Bioinformatics*.

[74] Taylor R. C. (2010). An overview of the Hadoop/MapReduce/HBase framework and its current applications in bioinformatics. *BMC Bioinformatics*.

[75] O'Connor B. D., Merriman B., Nelson S. F. (2010). SeqWare Query Engine: storing and searching sequence data in the cloud. *BMC Bioinformatics*.

[76] Wang S., Pandis I., Wu C. (2014). High dimensional biological data retrieval optimization with NoSQL technology. *BMC Genomics*.

[77] Bonnici V., Russo F., Bombieri N., Pulvirenti A., Giugno R. (2014). Comprehensive reconstruction and visualization of non-coding regulatory networks in human. *Frontiers in Bioengineering and Biotechnology*.

[78] Shannon P., Markiel A., Ozier O. (2003). Cytoscape: a software Environment for integrated models of biomolecular interaction networks. *Genome Research*.

[79] Cotillard A., Kennedy S. P., Kong L. C. (2013). Dietary intervention impact on gut microbial gene richness. *Nature*.

[80] CEGEDIM http://www.cegedim.fr/.

[81] Boulakia S. C., Lair S., Stransky N. (2004). Selecting biomedical data sources according to user preferences. *Bioinformatics*.

[82] Pruitt K. D., Brown G. R., Hiatt S. M. (2014). RefSeq: an update on mammalian reference sequences. *Nucleic Acids Research*.

[83] Blattner F. R., Plunkett G., Bloch C. A. (1997). The complete genome sequence of Escherichia coli K-12. *Science*.

[84] Yandell M., Ence D. (2012). A beginner's guide to eukaryotic genome annotation. *Nature Reviews Genetics*.

[85] Almeida L. G. P., Paixão R., Souza R. C. (2004). A System for Automated Bacterial (genome) Integrated Annotation—SABIA. *Bioinformatics*.

[86] Aziz R. K., Bartels D., Best A. (2008). The RAST Server: rapid annotations using subsystems technology. *BMC Genomics*.

[87] Vallenet D., Engelen S., Mornico D. (2009). MicroScope: a platform for microbial genome annotation and comparative genomics. *Database*.

[88] Tcherepanov V., Ehlers A., Upton C. (2006). Genome annotation transfer utility (GATU): rapid annotation of viral genomes using a closely related reference genome. *BMC Genomics*.

[89] Otto T. D., Dillon G. P., Degrave W. S., Berriman M. (2011). RATT: rapid annotation transfer tool. *Nucleic Acids Research*.

[90] Galeote V., Bigey F., Devillers H., Neuveglise C., Dequin S. (2013). Genome sequence of the food spoilage yeast *Zygosaccharomyces bailii* CLIB 213^T^. *Genome Announcements*.

[91] Freel K. C., Sarilar V., Neuveglise C., Devillers H., Friedrich A., Schacherer J. (2014). Genome sequence of the yeast *Cyberlindnera fabianii* (*Hansenula fabianii*). *Genome Announcements*.

[92] Neuvéglise C., Marck C., Gaillardin C. (2011). The intronome of budding yeasts. *Comptes Rendus Biologies*.

[93] Rutherford K., Parkhill J., Crook J. (2000). Artemis: sequence visualization and annotation. *Bioinformatics*.

[94] Jiang H., Wang F., Dyer N. P., Wong W. H. (2010). CisGenome browser: a flexible tool for genomic data visualization. *Bioinformatics*.

[95] Toedling J., Ciaudo C., Voinnet O., Heard E., Barillot E. (2010). Girafe—an R/Bioconductor package for functional exploration of aligned next-generation sequencing reads. *Bioinformatics*.

[96] Nolan D., Lang D. T. (2012). Interactive and animated scalable vector graphics and R data displays. *Journal of Statistical Software*.

[97] Robinson J. T., Thorvaldsdóttir H., Winckler W. (2011). Integrative genomics viewer. *Nature Biotechnology*.

[98] Westesson O., Skinner M., Holmes I. (2013). Visualizing next-generation sequencing data with JBrowse. *Briefings in Bioinformatics*.

[99] Hou H., Zhao F., Zhou L. L. (2010). MagicViewer: integrated solution for next-generation sequencing data visualization and genetic variation detection and annotation. *Nucleic Acids Research*.

[100] Arner E., Hayashizaki Y., Daub C. O. (2010). NGSView: an extensible open source editor for next-generation sequencing data. *Bioinformatics*.

[101] Shen L., Shao N., Liu X., Nestler E. (2014). Ngs.plot: quick mining and visualization of next-generation sequencing data by integrating genomic databases. *BMC Genomics*.

[102] Fiume M., Williams V., Brook A., Brudno M. (2010). Savant: genome browser for high-throughput sequencing data. *Bioinformatics*.

[103] Milne I., Bayer M., Cardle L. (2010). Tablet—next generation sequence assembly visualization. *Bioinformatics*.

[104] Riba-grognuz O., Keller L., Falquet L., Xenarios I., Wurm Y. (2011). Visualization and quality assessment of de novo genome assemblies. *Bioinformatics*.

[105] Kent W. J. (2002). BLAT—the BLAST-like alignment tool. *Genome Research*.

[106] Murrell P., Potter S. (2013). Advanced SVG graphics from R.

[107] Gentleman R. C., Carey V. J., Bates D. M. (2004). Bioconductor: open software development for computational biology and bioinformatics. *Genome Biology*.

[108] Gómez J., García L. J., Salazar G. A. (2013). BioJS: an open source JavaScript framework for biological data visualization. *Bioinformatics*.

[109] Bostock M., Ogievetsky V., Heer J. (2011). D^3^ data-driven documents. *IEEE Transactions on Visualization and Computer Graphics*.

[110] http://www.zoomdata.com/.

[111] Goecks J., Eberhard C., Too T., Nekrutenko A., Taylor J. (2013). Web-based visual analysis for high-throughput genomics. *BMC Genomics*.

[112] Google Genomics https://cloud.google.com/genomics/.

[113] Codd E. F. Relational completeness of database sublanguages.

[114] Ng P. C., Kirkness E. F. (2010). Whole genome sequencing. *Methods in Molecular Biology*.

